# Img2Variety: Image-based intraspecific varieties identification across the whole growth period

**DOI:** 10.1016/j.plaphe.2025.100151

**Published:** 2025-12-18

**Authors:** Yongrong Cao, Hong Luo, Rongye Ye, Lun Li, Zishan Wu, Dongmei Tian, Shuhui Song

**Affiliations:** a National Genomics Data Center, China National Center for Bioinformation, Beijing, 100101, China; b Beijing Institute of Genomics, Chinese Academy of Sciences, Beijing, 100101, China; c Sino-Danish College, University of Chinese Academy of Sciences, Beijing, 100049, China; d University of Chinese Academy of Sciences, Beijing, 100049, China

**Keywords:** Image augmentation, Image classification, Intraspecific varieties identification, Transfer learning, Convolution neural network

## Abstract

Accurate identification of crop varieties across growth stages is fundamental for material verification and trial management, providing a reliable basis for subsequent performance evaluation and elite accession selection in breeding programs. However, it remains challenging to differentiate intraspecific varieties due to subtle morphological variations among closely related accessions. Here, we present Img2Variety, a novel convolutional neural network (CNN)-based framework for crop accession identification from whole-plant images. Img2Variety builds on transfer learning by fine-tuning pre-trained CNNs. It is designed to adapt to plant datasets with a large number of accessions but limited samples per accession, thereby improving generalization across diverse accessions. To enrich feature diversity, we propose a novel growth stage and multi-view mixed augmentation (GMMA) strategy that leverages variation in viewing angles and developmental stages to promote feature learning. We also employ an adaptive cross-entropy (ACE) loss that emphasizes misclassified samples during training to improve identification performance. Img2Variety was evaluated using six CNN backbones on two datasets: one comprising 11,170 RGB images of 93 rice (*Oryza sativa*) accessions throughout the entire growth period, and another containing 5,599 RGB images of 224 maize (*Zea mays*) inbred lines across nine growth stages. Img2Variety achieved a peak accuracy of 88.66 % for rice and 79.95 % for maize, with an average relative improvement of 86.30 % over six baseline methods on the maize dataset. Notably, it exceeded 80.22 % accuracy for pre-heading rice and the maize tenth-leaf stage. These results highlight Img2Variety's effectiveness in crop variety identification and its potential for early-stage crop management. A web-based implementation is freely accessible at https://ngdc.cncb.ac.cn/opia/img2variety.

## 1 Introduction

Precision breeding plays a crucial role in ensuring global food security and advancing agricultural sustainability [1]. Crops exhibit vast genetic diversity, with rice alone comprising over 500,000 accessions cataloged worldwide (available at: https://www.genesys-pgr.org/c/rice). These accessions vary significantly in critical agronomic traits such as yield potential, disease resistance, and environmental adaptability. However, accurately identifying and differentiating accessions during early developmental stages remains a major challenge. The precise identification of crop varieties not only assists farmers in selecting optimal accessions to enhance productivity but also plays a vital role in resource conservation [2], accession management [3], and advancements in breeding techniques [4]. Traditional classification methods primarily rely on knowledgeable breeders [5,6] or molecular marker assessments [4,7,8]. While these approaches are effective, they are labor-intensive, time-consuming, and require specialized equipment or expertise. Furthermore, traditional methods often struggle with fine-grained classification, which requires distinguishing accessions or subspecies within a single crop species. They also face challenges in early-stage identification, where accessions must be recognized during initial growth stages, such as the pre-heading stage in rice or up to the tenth-leaf stage in maize. These capabilities are crucial for shortening breeding cycles and achieving precise breeding objectives.

Recent advancements in deep learning and computer vision have enabled promising breakthroughs in plant species classification and accession identification. A growing body of research has explored the use of CNNs for analyzing plant leaf images to achieve non-destructive, efficient, and automated identification. For instance, Ganguly et al. [9] proposed BLeafNet, a CNN-based model incorporating the Bonferroni mean operator to improve classification accuracy across multiple plant leaf datasets. Similarly, other studies [[10], [11], [12]] have demonstrated the effectiveness of CNNs in distinguishing between plant accessions using visual leaf characteristics. One notable example is MFCIS [13], which achieved an accuracy of 83.52 % in identifying 88 sweet cherry cultivars from over 5,000 leaf images, and 91.40 % accuracy in classifying 100 soybean cultivars from 5,000 leaf images across five growth stages. These approaches have proven valuable for fine-grained classification and show great potential for supporting crop breeding and germplasm management. Despite their promise, leaf-based CNN methods are limited by their reliance on single-organ and fixed-viewpoint images. This hinders the modeling of phenotypic variation across developmental stages and viewpoints, which is crucial for identifying similar varieties with limited data [14,15].

Transfer learning [16] is a transformative approach that enables the reuse of knowledge from source tasks to improve model performance on target tasks, especially in data-scarce scenarios. Leveraging large-scale datasets like ImageNet [17] reduces the reliance on extensive annotations while enhancing model efficiency and generalization. Classical CNN architectures, such as VGG19 [18], ResNet18 [19], and DenseNet121 [20], have been widely used as backbones in transfer learning pipelines across diverse fields. In agricultural applications, transfer learning has proven instrumental in addressing key challenges, such as the precise detection of plant diseases [11,21,22] and the identification of varieties [[23], [24], [25]]. These studies collectively show that transfer learning provides a powerful strategy for improving model performance when annotated agricultural data are limited. Furthermore, image augmentation methods—like mixup, scaling, and rotation—enhance data diversity and model robustness [26]. Together, these techniques provide a robust methodological foundation for developing accurate generalizable image-based crop variety identification systems.

In this study, we propose Img2Variety, a deep-learning framework optimized for intraspecific crop variety identification, i.e., distinguishing varieties within the same species. The pipeline leverages transfer learning to better handle the limited scale and phenotypic diversity of plant image datasets. To enhance data diversity, we design a growth stage and multi-view mixed augmentation (GMMA) strategy that mixing images from different viewpoints and adjacent growth stages, expanding the training distribution while maintaining label consistency. To mitigate misclassification caused by ambiguous phenotypes, we incorporate an adaptive cross-entropy (ACE) loss [27], which increases the contribution of misclassified samples to the overall loss, thereby guiding the model to focus more on hard-to-classify case. We validated Img2Variety on two datasets—spanning 93 rice accessions and 224 maize inbred lines—across multiple growth stages, and benchmarked its performance against six widely used CNN architectures.

## 2 Materials and methods

### 2.1 Image datasets and data processing

We analyzed image datasets from two species with varying accession sizes and image counts: (1) the Rice (*Oryza sativa* L.) dataset [28] [29] and (2) the Maize (*Zea mays* L.) dataset [30]. The rice dataset contains a total of 11,170 raw RGB images, and the maize dataset contains 5,599 raw RGB images. Two datasets associated with annotations have been curated and archived in an open plant image archive database (OPIA, https://ngdc.cncb.ac.cn/opia/) [29] with the rice dataset available as “WGSR” and the maize dataset as “WGSM”.

**Fig. 1:**
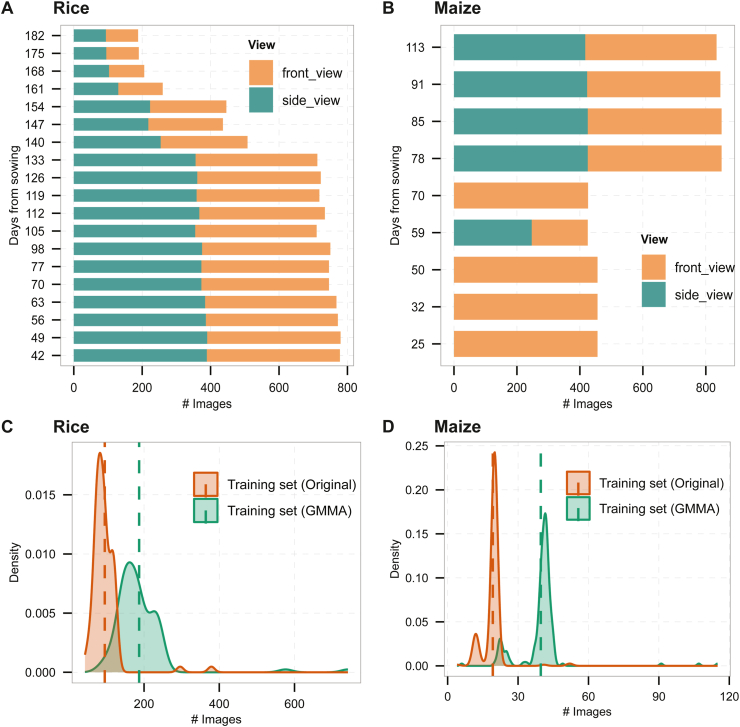
Distribution of the original and GMMA processed image data for rice and maize. (A) Images collected at different growth stages for 93 rice accessions (42–182 days after sowing) from two viewing angles: front view and side view. (B) Images of 224 maize lines at nine growth stages (25–133 days after sowing), the data also includes front view and side view. (C) Density plot of the number of images per accession in the rice training dataset. The dashed line represents the average number of original and GMMA training images for all rice accessions, respectively. (D) Density plot of the number of images per accession in the maize training dataset. The dashed line represents the average number of original and GMMA training images for all maize inbreed lines, respectively.

The rice dataset was obtained from the Institute of Genetics and Developmental Biology, Chinese Academy of Sciences, in Beijing. A total of 93 rice accessions (61 japonica and 32 indica) were grown in a controlled greenhouse environment. According to the dataset source [28], seed germination was performed at 25–32 °C for approximately 24 h, followed by bud airing at 20 °C for about 20 h before transplantation into the greenhouse. The greenhouse was maintained under controlled environmental conditions, with stable temperature and humidity levels suitable for rice cultivation, as reported in the source dataset. Imaging was conducted under consistent artificial lighting conditions within the greenhouse, without natural light interference. All accessions were monitored from 42 to 182 days after sowing, with images captured approximately every seven days (Fig. 1A). The dataset consisted of both front-view and side-view RGB images of growing plants, captured by a phenotyping facility (Scanalyzer, LemnaTec GmbH, Germany) at a resolution of 2056 × 2452 pixels.

The Rice dataset provides statistical data of images at two levels: subspecies and accession (Table S1). The original datasets were randomly partitioned into training and testing sets with an 80 % and 20 % distribution, respectively. Fig. S1A displays selected samples from one rice accession (Longjing31), illustrating the phenotypic variations observed at different stages throughout the entire growth period. To reduce the impact of complex background elements on CNN models’ performance, the original images were cropped to focus solely on the plants using an in-house python program. Fig. S1B shows the images after cropping, followed by resizing to standardized dimensions. To strike a balance between model efficiency and computational resource consumption, the images were resized to 360 × 480 pixels.

The Maize dataset [30] comprises 224 representative maize inbred lines, which encompasses local and elite lines, integrating genetic resources from temperate, subtropical, and tropical regions, all cultivated in greenhouses in Hebei Province, China [30]. The images were collected at a resolution of 2454 × 2056 and were documented at various developmental stages throughout their growth (S1-S9 corresponding to images captured at 25, 32, 50, 59, 70, 78, 85, 91, and 113 days after sowing) (Fig. 1B). As part of the preprocessing pipeline, all images were resized to 409 × 342 pixels.

### 2.2 Growth stage and multi-view mixed augmentation (GMMA)

Data augmentation has emerged as an essential technique in deep learning for increasing the diversity of training datasets and improving model generalization. In image classification tasks, augmentation strategies are commonly used to artificially expand datasets by introducing variations to the original images [26]. Hao et al. demonstrated that augmenting data through transfer learning significantly boosts performance in image-text retrieval tasks [31]. This approach inspired the development of our GMMA method aimed at leveraging spatial and temporal variations within the rice accession dataset.

Algorithm 1 GMMA

**Table:** 

Input: folder (image dataset path), λ (mixing coefficient, 0.5)
Output: Mixed images saved to folder
1. Define function mixgen (${image}_{i}$, $i{mage}_{j}$, λ):
-Returns mixed image: $I_{mixed}=\lambda \cdot I_{i}+\left(1-\lambda \right)I_{j}$
# Define image file names based on the naming rule: "accession_tag_date_viewpoint.png"
2. Group images by accession tags
-Group images based on their accession tags (e.g., R0001, R0002, etc.)
3. For each accession group G in files:
-If |G|>1: # If there are multiple images in the group
For each image pair ($I_{i}$, $I_{j}$) in G:
-Generate mixed image:
# Same viewpoint with adjacent sampling date
$I_{mixed,temporal}$ ← mixgen($I_{temp}^{t}$, $I_{temp}^{t+1}$, λ)
# Same sampling date with different viewpoints
$I_{mixed,viewpoint}$ ← mixgen($I_{view}^{f}$, $I_{view}^{s}$, λ)
# Different viewpoints with adjacent sampling date
$I_{mixed,view-stage}$ ← mixgen($I_{temp,view}^{t,f}$, $I_{temp,view}^{t+1,s}$, λ)
-Save $I_{mixed}$ with a new mixed filename

**Fig. 2:**
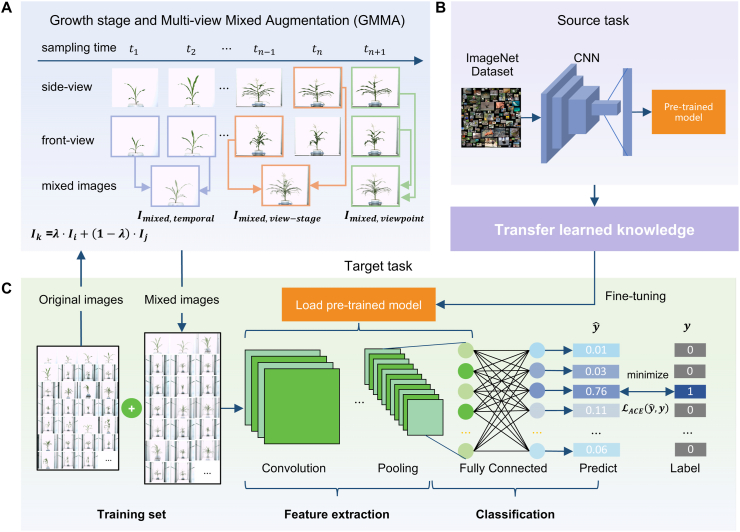
Workflow of Img2Variety, an image-based deep learning framework for crop accession identification. **(**A) Illustration of the GMMA strategy. Given two images, $I_{i}$ and $I_{j}$, a new image $I_{k}$ is generated through interpolation. GMMA includes three types of mixing: (I) temporal mixing of the same viewpoint at adjacent sampling time points, resulting in image $I_{mixed,temporal}$ (indicated by blue arrows); (II) viewpoint mixing at the same sampling time point, resulting in image $I_{mixed,viewpoint}$ (indicated by green arrows); and (III) view-stage mixing of different viewpoints across adjacent time points, resulting in image $I_{mixed,view-stage}$ (indicated by orange arrows). (B) Transfer learning phase I: a CNN is pre-trained on a large-scale source task. (C) Transfer learning phase II: the entire CNN is fine-tuned using both the original and GMMA-augmented images to perform crop variety identification on the target task.

The GMMA algorithm generates enhanced images by incorporating temporal and spatial dimensions. The pseudocode is presented in Algorithm 1, and the GMMA schematic representation of the three types of mixing is shown in Fig. 2A. Specifically, temporal mixing combines images captured at consecutive times of the accession representing growth progression. Spatial mixing combines images taken from different viewpoints of the accession on the same date, capturing variations in appearance due to perspective changes. Spatiotemporal mixing fuses images of the same rice accession taken at consecutive time points but from different angles (spatial perspectives), capturing dynamic growth changes alongside perspective diversity. Let $I_{temp}^{t}$ and $I_{temp}^{t+1}$ represent two images from consecutive time points t and t+1. $I_{view}^{f}$ and $I_{view}^{s}$ represent spatial images of the accession taken on the same date from front-view and side-view angles. $I_{temp,view}^{t,f}$ represents the front-view image at time t, and $I_{temp,view}^{t+1,s}$ represents the side-view image at the subsequent time t+1. The mixed images are formulated as follows:

(1) $$I_{mixed,temporal}=\lambda \cdot I_{temp}^{t}+\left(1-\lambda \right)\cdot I_{temp}^{t+1}\text{,}$$

(2) $$I_{mixed,viewpoint}=\lambda \cdot I_{view}^{f}+\left(1-\lambda \right)\cdot I_{view}^{s}\text{,}$$

(3) $$I_{mixed,view-stage}=\lambda {\cdot I}_{temp,view}^{t,f}+\left(1-\lambda \right){\cdot I}_{temp,view}^{t+1,s}\text{,}$$

where λ is the mixing coefficient (typically set to 0.5) that controls the contribution of each input image to the resulting mixed image.

We applied GMMA to accessions with available temporal and multi-view images to increase training diversity (Table S1). After augmentation, the rice training set expanded from 8895 to 17,391 images, increasing the average number per accession from 96 to 187 (Fig. 1C). This increase comprised 3521 images generated by viewpoint mixing, 1416 by temporal mixing, and 3559 by view-stage mixing. For maize, the training set grew from 4307 to 8887 images, with the average rising from 19 to 40 images per accession (Fig. 1D). The additional images included 983 produced by viewpoint mixing, 2474 by temporal mixing, and 1123 by view-stage mixing.

### 2.3 The architectural of Img2Variety and backbone selection

Img2Variety employs the pre-trained models, capitalizing on their robust feature extraction abilities. This approach significantly boosts model performance by integrating our GMMA techniques with adaptive cross-entropy loss (ACE Loss) functions (Fig. 2). Specifically, we leverage transfer learning [16] utilizing pre-trained convolutional layers to extract hierarchical features from images. Six models trained on the extensive ImageNet dataset extract generalizable features across various levels. In the lower layers, these networks recognize basic features like edges, textures, and colors, which are fundamental components of visual data. The intermediate layers detect more intricate patterns, including shapes and object parts, enhancing the model's comprehension of object structure. Ultimately, the upper layers capture semantic representations, allowing the network to differentiate between high-level categories. To better adapt these hierarchical representations to the domain-specific task of variety identification, we employ full fine-tuning of all model parameters rather than freezing earlier layers. This strategy allows the network to adjust both low- and high-level features in response to phenotypic cues, leading to improved task-specific performance.

The six CNN backbones—VGG19 [18], ResNet18 [19], MobileNetV2 [32], InceptionV3 [33], EfficientNet [34], and DenseNet121 [20]—were selected to represent diverse architectural designs, spanning different depths, parameter sizes, and computational complexities. Their model sizes and GPU memory usage are summarized in Table S2 and S3, providing a basis for evaluating trade-offs between accuracy and efficiency in fine-grained crop classification tasks. Our specific tasks focus on rice accession identification (93 categories) and maize accession identification (224 categories), we train separate classification models based on six classical CNNs. The methodology for employing these models is detailed below:

We modified the final fully connected layer to correspond with the number of classes in our dataset. This multi-model strategy enabled us to evaluate the effectiveness of various architectures and determine the optimal model for our classification objectives. In our classification framework, we initiated the process by utilizing the standard cross-entropy loss function to assess model performance. The cross-entropy loss [35] serves as a widely accepted metric that effectively measures the disparity between predicted probabilities and actual class labels, making it applicable for both binary and multi-class classification tasks. The cross-entropy loss function is mathematically represented as follows:

(4) $$L_{CE}=-\frac{1}{N}\sum_{i=1}^{N}\sum_{j=1}^{C}y_{ij}\cdot \log\;{\hat{y}}_{ij}\text{,}$$

where N is the total number of images in the dataset, C is the total number of categories, $y_{ij}$ represents the true label for sample i and class j, ${\hat{y}}_{ij}$ denotes the predicted probability that image i belongs to class j. This loss function measures the dissimilarity between the predicted class probabilities and the actual class labels. However, we observed that standard cross-entropy loss tends to treat all samples equally during training, which may limit the model's ability to focus on misclassification instances. In fine-grained classification tasks—such as rice and maize accession identification—certain samples exhibit high visual similarity and are more prone to misclassification.

To tackle this challenge, we devised a custom adaptative cross entropy loss (ACE Loss), which adjusts the standard cross entropy loss by incorporating additional weights for misclassified samples. The implementation of the ACE Loss is detailed as follows:

(5) $${L_{ACE}=L}_{CE}-\frac{1}{N}\alpha \sum_{i=1}^{N}\left(∼{correct}_{i}\right)\sum_{j=1}^{C}y_{ij}\cdot \log\;{\hat{y}}_{ij}\text{,}$$

where α is a hyperparameter that controls the additional weight for misclassified samples, and ${correct}_{i}$ is an indicator function that equals 1 if the prediction for sample i is correct and 0 otherwise.

### 2.4 Hardware setup and training configuration for intraspecific crop identification

The experiments were implemented under the following hardware configuration: Hygon C86 7285 32-core Processor, 32 GB memory, and two graphics cards NVIDIA Tesla V100S PCIe 32 GB. All models were trained using the backpropagation method and optimized with Stochastic Gradient Descent (SGD) algorithm. The learning rate was configured to 0.01, with a momentum value of 0.9. All models were executed using the PyTorch framework (version 1.12.0), with a batch size of 64, running on CUDA 11.4 to leverage GPU acceleration. Python version 3.8 was used for this experiment, and additional libraries installed for data analysis and visualization included scikit-learn 1.3.2, seaborn 0.13.2, and OpenCV-python 4.10.0.84. In both the rice and maize accession identification tasks, the models were trained for 150 epochs. The rice subspecies classification task, being a simpler task, was trained for 50 epochs. We adopted random horizontal flipping of image techniques combined with GMMA during training. Each image had a 50 % probability of being flipped, which helped the model generalize better by learning features of plants from different orientations. Detailed parameter settings for rice and maize accession identification using the six backbone networks are provided in Table S4 and S5.

### 2.5 Evaluation metrics for image classification performance

In image classification tasks, several essential evaluation metrics are frequently utilized to assess model performance. All metrics except accuracy are macro-averaged across all accessions. These metrics include accuracy, precision, recall, and $F_{1-score}$, the formulas are as follows:

(6) $$Accuracy=\sum_{i=1}^{C}\frac{{TP}_{i}}{N}\text{,}$$

(7) $$Precision=\frac{1}{C}\sum_{i=1}^{C}\frac{{TP}_{i}}{{TP}_{i}+{FP}_{i}}\text{,}$$

(8) $$Recall=\frac{1}{C}\sum_{i=1}^{C}\frac{{TP}_{i}}{{TP}_{i}+{FN}_{i}}\text{,}$$

(9) $$F_{1-score}=\frac{1}{C}\sum_{i=1}^{C}2\times \frac{{Precision}_{i}\times {Recall}_{i}}{{Precision}_{i}+{Recall}_{i}}\text{,}$$

where C denotes the number of accessions, N represents the total number of testing images. For a given accession: TP (True Positive) refers to the number of images that are correctly identified as belonging to a specific accession. FP (False Positive) refers to the number of images that are incorrectly predicted as belonging to a specific accession, while they actually belong to another. FN (False Negative) denotes the number of images that truly belong to that accession but are incorrectly predicted as not belonging to it.

### 2.6 Baseline traditional machine learning classifiers

We implemented four traditional machine learning models—Naïve Bayes, Decision Tree, Support Vector Machine (SVM), and K-Nearest Neighbors (KNN)—to establish baseline performance for the subspecies- and accession-level rice classification task. These methods have been widely used in plant image analysis due to their efficiency and interpretability [[36], [37], [38]]. For each image, several complementary descriptors were extracted. Global shape information was captured using Hu invariant moments derived from image moments [39]. Textural cues were obtained using a subset of Haralick features, specifically the contrast statistic, which was computed from grayscale images at multiple distances and orientations [40]. Local texture patterns were summarized using uniform Local Binary Patterns (LBP) with a radius of 3 pixels and 24 sampling points, followed by a 26-bin histogram [41]. In addition, overall color characteristics were represented by the mean hue, saturation, and value components in HSV color space. All features were standardized using z-score normalization and classified using the scikit-learn [42] implementation. The same train/test partitions as the CNN models were used to ensure a consistent basis for comparison.

### 2.7 Gradient-weighted Class Activation Mapping (Grad-CAM) visualization

To interpret the model's decision-making process in variety identification, we employed Gradient-weighted Class Activation Mapping (Grad-CAM) [43]. Grad-CAM generates a localization map that highlights the most influential regions of an input image for predicting a specific variety.

Given a predicted variety v , Grad-CAM computes the gradient of the variety score $\left(\text{logit}\right)\;y^{v}$ with respect to the activation feature maps $A^{k}$ of a last convolutional layer, where $A^{k}$ denotes the k-th channel feature map. These gradients reflect the sensitivity of the variety prediction to each spatial location in the feature maps. The sensitivity weight $\alpha_{k}^{v}$ for each feature map is then obtained by applying global average pooling to the gradients:

(10) $$\alpha_{k}^{v}=\frac{1}{F}\sum_{i}\sum_{j}\frac{\partial y^{v}}{\partial A_{ij}^{k}}\text{,}$$

where $A_{ij}^{k}$ represents the activation at spatial position (i,j) of the k-th feature map, and F=H×W is the total number of spatial locations. The weighted feature maps are then combined linearly and passed through a ReLU activation to produce the final Grad-CAM heatmap:

(11) $$L_{Grad-CAM}^{v}=ReLU\left(\sum_{k}\alpha_{k}^{v}A^{k}\right)\text{,}$$

where ReLU(x)=max(0,x) ensures that only positive contributions are preserved. The resulting heatmap $L_{Grad-CAM}^{v}$ identifies the regions in the input image that are most relevant to the prediction of variety v. Overlaying this heatmap onto the original image enables visual interpretation of which plant regions the model focuses on during classification.

## 3 Results

### 3.1 Img2Variety outperforms CNN baseline models for accession identification

**Fig. 3:**
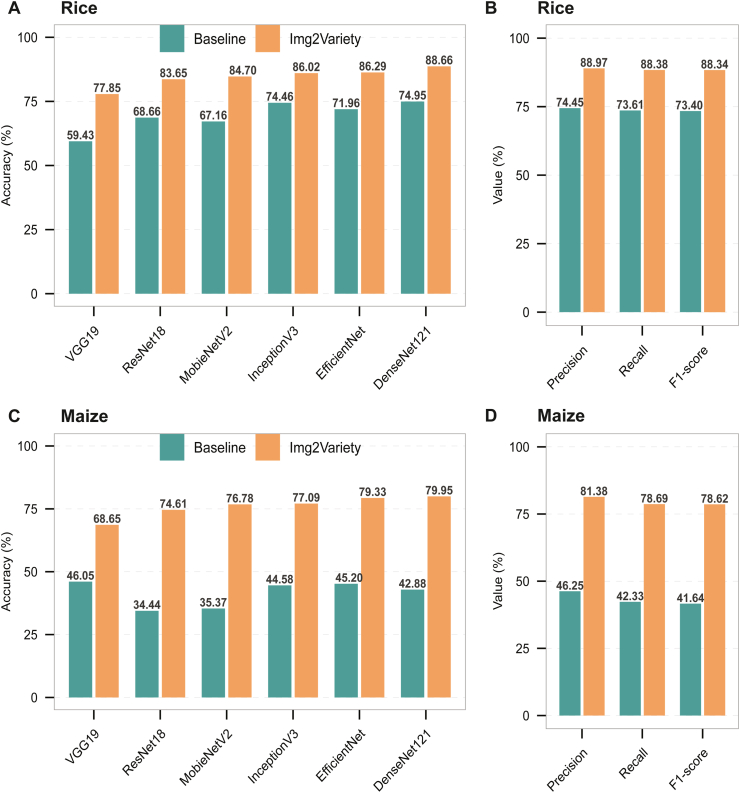
Comparison of accession identification performance between Img2Variety and the corresponding baseline model on rice and maize datasets. (A, C) Identification accuracy comparison for the rice (A) and maize (C) datasets. (B, D) Precise, recall, and F1-score of the optimal Img2Variety based on DenseNet121 comparison for the rice (B) and maize (D) datasets.

Img2Variety consistently outperformed the six baseline CNN architectures on both rice and maize datasets. On the rice dataset, which includes 93 accessions, Img2Variety achieved an average relative accuracy improvement of 22.45 % over the baselines (Fig. 3A). Notably, Img2Variety exhibited a remarkable 30.99 % relative increase in accuracy over the VGG19 baseline, rising from 59.43 % to 77.85 %. The best-performing Img2Variety model, based on DenseNet121, achieved an accuracy of 88.66 %, along with corresponding enhancements in precision, recall, and F1-score (Fig. 3B, Table S2).

For the maize dataset, we implemented similar training and testing procedures to evaluate the performance of Img2Variety in identifying 224 inbred lines. Img2Variety achieved an accuracy ranging from 68.65 % to 79.95 % for maize variety identification, with an average relative accuracy increase of nearly 87.00 %, demonstrating significant improvement over the six benchmark models (Fig. 3C). The best-performing Img2Variety model, based on DenseNet121, enhanced accuracy from 42.88 % to 79.95 %, reflecting a relative increase of 86.45 %, and achieved an average improvement of 83.56 % in precision, recall, and F1-score (Fig. 3D, Table S3).

Our results demonstrate that Img2Variety consistently outperforms widely adopted image classification backbones for intraspecific variety identification. Furthermore, Img2Variety shows strong generalization capability, achieving robust performance across crop datasets with varying numbers of accessions.

### 3.2 Performance of Img2Variety across growth stages

**Fig. 4:**
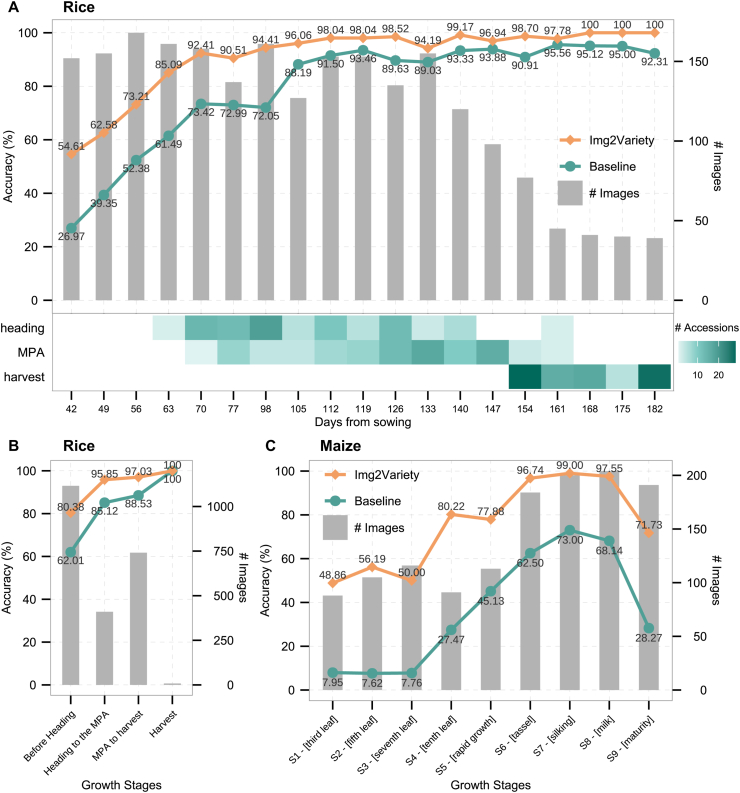
Accuracy of Img2Variety and baseline DenseNet121 models for rice and maize variety identification across different growth stages. (A) Accuracy across weekly sampling intervals days from sowing for identifying 93 rice accessions. The lower panel illustrates the distribution of the 93 rice accessions at three developmental milestones: heading, maximum projected area (MPA), and harvest stage. (B) Accuracy distribution across four key phenological stages for rice: pre-heading, heading to maximum projected area (MPA), MPA to pre-harvest, and harvest. (C) The comparative accuracy assessment for identifying 224 maize lines across nine growth stages. For all panels, the secondary y-axis (bar plots) indicates the number of test images available at each respective stage.

To further evaluate the capability of Img2Variety in recognizing plant varieties at different growth stages, we assessed its classification accuracy using test-set images captured at various days from sowing. Img2Variety exhibited significant improvements in variety identification accuracy compared to the baseline DenseNet121 model across whole development periods (Fig. 4A). At the seedling stage (e.g., 42 days post-sowing), a period characterized by minimal visual differences among rice varieties, the baseline model achieved only 26.97 % accuracy, while Img2Variety reached 54.61 %, representing an almost twofold improvement. With increasing days after sowing, Img2Variety's variety identification accuracy improved steadily and eventually reached 100 %. Considering the variation in growth periods among different cultivars, we further grouped the test images into four major phenological stages: (1) pre-heading, (2) heading to maximum projected area (MPA), (3) MPA to pre-harvest, and (4) harvest. Img2Variety achieved over 80.00 % accuracy during the early (pre-heading) stage and surpassed 95.85 % accuracy from heading through maturity, demonstrating robust performance even during early phenological phases (Fig. 4B).

Fig. 4C presents a comparison of maize variety identification accuracy between the baseline DenseNet121 and Img2Variety models across nine defined growth stages. The baseline model exhibited relatively low accuracy in the early stages (S1–S3), peaking at 73 % during the tasseling stage (S6), but showed a marked decline by the maturity stage (S9). In contrast, Img2Variety consistently maintained higher accuracy, with particularly notable improvements in the mid-to-late stages (S4–S8), reaching a peak of 99 % during the silking stage (S7).

These results highlight Img2Variety's enhanced ability to extract discriminative features during early development and to leverage distinct morphological characteristics in later stages. The framework notably reduced misclassifications, especially at critical phenological points such as tasseling and heading. This improved classification performance is vital for accurate crop variety identification and offers practical insights for modern agriculture, particularly in supporting informed decision-making during key growth stages.

### 3.3 Superior performance of Img2Variety on rice subspecies classification

We evaluated the performance of Img2Variety on classifying the two subspecies, indica and japonica, using six CNN backbones (Table S6, Fig. S2). Across all architectures, Img2Variety achieved consistently high accuracy, ranging from 94.51 % to 96.97 %, accompanied by similarly strong precision, recall, and F1-scores. We also compared Img2Variety with four traditional machine learning classifiers evaluated on the same task (Table S7). These classical models produced considerably lower accuracies, which varied between 52.66 % and 80.35 %, and also exhibited reduced stability across evaluation metrics. This performance gap underscores that subspecies classification, while partly solvable using handcrafted features, still benefits significantly from deep learning–based representation learning. These results confirm that subspecies discrimination is a relatively accessible task and serves as a reliable benchmark for evaluating Img2Variety capability.

### 3.4 Improved attention localization across growth stages in rice and maize

**Fig. 5:**
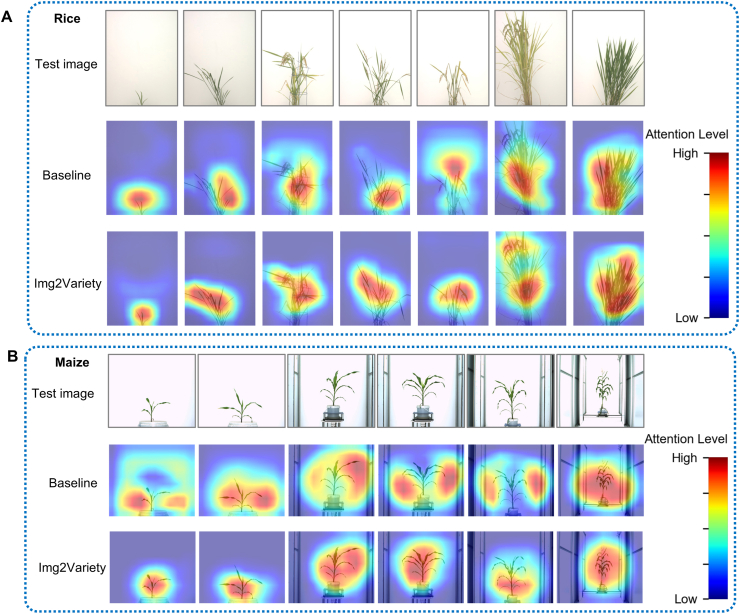
Comparison of attention maps between the baseline (DenseNet121) and Img2Variety on rice (A) and maize (B) across different growth stages. Each group consists of three rows: the first row shows the test images, the second row presents the Grad-CAM attention maps generated by the baseline model, and the third row shows the attention maps from Img2Variety. The attention regions are color-coded from red (high attention) to blue (low attention).

To explore the interpretability of our method and whether it consistently focuses on semantically meaningful regions across growth stages. We visualized Gradient-weighted Class Activation Mapping (Grad-CAM) attention maps on test samples from the rice and maize datasets (Fig. 5) [43]. The visualization compares the baseline model and our proposed method across multiple growth stages.

In the rice dataset (Fig. 5A), our method consistently concentrates on relevant plant regions across different growth stages. For seedling-stage images (first two columns), Img2Variety highlights the entire plant body, including both leaves and stems. In contrast, the baseline model often shows scattered attention or focuses on the background. In the later stages (last five columns), where panicle structures become visually apparent, Img2Variety's attention shifts toward the panicle—a key morphological trait used in manual cultivar identification—demonstrating a biologically meaningful focus. This stage-specific shift aligns well with agronomic knowledge and reinforces the interpretability of the model.

In the maize dataset (Fig. 5B), similar patterns are observed. The baseline model frequently allocates high attention to irrelevant regions such as plant borders and background clutter. Our method, however, consistently focuses on the stalk and leaf regions. This demonstrates that the model has effectively learned to localize the main crop body and ignore distractors, thereby contributing to improved generalization and robustness.

Overall, Img2Variety significantly improves identification accuracy. This improvement can be attributed to the model's ability to focus on growth stage-specific regions and key plant structures, such as the panicle, which serve as crucial visual cues for agronomic experts [[44], [45], [46]].

### 3.5 Online identification platform based on Img2Variety

To facilitate practical applications of our framework, we developed an online identification platform powered by Img2Variety for crop variety identification. The current version supports rice and maize, reflecting the datasets and training scenarios described in this study. Users can upload a single RGB image of an individual plant—captured at any growth stage and from any viewpoint—or submit a compressed archive (e.g., ZIP) containing multiple images, to receive real-time predictions of the corresponding variety. The web interface provides an intuitive user experience, including image upload, automatic preprocessing, and prediction output through a URL interface (https://ngdc.cncb.ac.cn/opia/img2variety). Upon clicking "Run", the system returns the predicted variety within seconds, providing a fast and accessible solution for image-based variety identification. To further enhance accessibility and performance, we have released the source code for local deployment. For optimal accuracy and speed—especially in high-throughput scenarios—we recommend running the pipeline on a high-performance GPU server.

## 4 Discussion

### 4.1 Feasibility and practicality of the Img2Variety framework

Accurate identification of crop accessions is crucial in modern agriculture and breeding programs [47], as it enables researchers and breeders to select varieties that are optimally adapted to specific environmental conditions and market needs. Traditional methods, such as expert-based visual recognition [48], often suffer from inconsistency and subjectivity. While molecular marker techniques, including DNA barcoding [8,49], offer more reliable, genetically based identification, they are often time-consuming and expensive. Recent advancements in artificial intelligence (AI)-based computer vision and high-throughput plant phenotyping have significantly improved the feasibility of using image-based approaches for crop variety identification [22,50,51]. In this study, we curated a robust RGB image dataset captured from two viewpoints across multiple growth stages, enabling the incorporation of both temporal and spatial phenotypic traits. To effectively leverage these spatiotemporal features, we further propose a novel augmentation strategy - GMMA, which significantly improves identification accuracy by integrating visual information across temporal and viewpoint. This approach demonstrates the practicality of using computer vision for automated, scalable, and accurate variety classification.

### 4.2 Ablation study on the key components of Img2Variety

We systematically evaluated the effects of transfer learning, ACE loss, and GMMA across five configurations and six backbone networks for both rice and maize (Table S8-S9). The results reveal three consistent patterns. First, transfer learning provides the largest single-component improvement across architectures and species, underscoring its importance when labeled data are limited. Second, GMMA introduces substantial additional gains, with consistently strong improvements observed across backbones. Third, ACE loss offers a smaller but reliable enhancement, particularly for more challenging or low-confidence samples.

To further quantify the individual effects of GMMA and ACE, we isolated these components using DenseNet121 (Table S10-S11). In rice, GMMA improves accuracy from 74.95 % to 84.35 % and ACE provides a modest but measurable gain. In maize, both components yield larger improvements, with GMMA and ACE increasing accuracy to 62.23 % and 52.63 %, respectively. Across all evaluations, the full model that integrates transfer learning, GMMA, and ACE achieves the best performance, reaching 88.66 % accuracy in rice and 79.95 % in maize with DenseNet121. These findings demonstrate that the three components are complementary and jointly enhance the robustness of Img2Variety.

To further evaluate the contribution of GMMA, we performed an ablation study examining three data augmentation strategies: temporal, multi-view, and their combination across both time and viewpoint dimensions. Experiments were performed using Img2Variety with DenseNet121, which previously yielded the best baseline performance. Starting from 8895 original RGB images, we generated additional training samples by constructing image pairs that met the criteria for each augmentation scheme. Classification results on the rice and maize datasets (Table S12 and S13) show that the combined spatiotemporal augmentation consistently outperformed single-dimensional approaches, indicating a synergistic effect in capturing complementary phenotypic cues across both temporal and viewpoint. The performance gain also reflects the benefit of increased sample diversity, which is known to improve generalization in deep learning [26,52,53]. Overall, each augmentation strategy contributed positively, and their integration within GMMA was the most effective, reinforcing the value of comprehensive spatiotemporal phenotypic information in image-based crop variety identification.

### 4.3 Phenotypic ambiguity and its impact on early-stage variety identification

During early growth stages—pre-heading in rice and before rapid growth in maize—crops show few distinct traits, making variety identification difficult. In rice, seedlings display high morphological similarity across accessions, with key differentiating traits such as panicle architecture and internodal spacing only emerging closer to heading. Similarly, in maize, features such as seedling height, leaf number, and juvenile leaf morphology prior to the tenth-leaf stage lack sufficient distinctiveness to support reliable classification, with clearer varietal differentiation typically observed during later vegetative and reproductive phases.

This phenotypic ambiguity during early development constrains model performance and increases misclassification risk. Our results demonstrate that incorporating temporal and multi-view image information enhances the model's ability to capture subtle visual cues within these early stages (Fig. 4A–C). Notably, we observed a marked improvement in early-stage accuracy for both rice and maize when enriched features were available, supporting the value of information continuity across developmental timepoints. This approach bridges the morphological gap between early and mature stages, narrowing the classification difference between early and late predictions. It also offers potential practical value for breeding programs that require early phenotypic screening.

### 4.4 From subspecies to accessions: challenges in fine-grained rice classification

Our results show a clear contrast between subspecies classification and accession-level identification (Table S7). Subspecies classification proves comparatively straightforward for Img2Variety, given that indica and japonica exhibit distinct morphological and genetic differences, enabling consistently high performance across CNN backbones. Conversely, accession-level classification constitutes a substantially more formidable challenge. Accessions within the same subspecies frequently exhibit highly analogous phenotypic characteristics, reflecting their closer genetic relationships and convergent morphological traits.

The misclassifications produced by Img2Variety at the accession level also show a biologically significant pattern (Fig. S3). The majority of errors occurred among accessions within the same subspecies rather than across subspecies boundaries. This observation implies that the model reliably discerns subspecies-level distinctions yet struggles to differentiate the subtle phenotypic variations that separate closely related accessions.

### 4.5 Limitations and further work

Our study also has its limitations, e.g., the datasets utilized in this research were collected under controlled conditions and within specific time frames, which may not fully reflect the variability in real-world agricultural environments. Environmental factors, such as variations in light, temperature, and soil conditions, can significantly impact the phenotypic traits of rice plants, posing additional challenges for accurate identification. So, we will focus on the generalization performance of deep learning models to improve their usability and trustworthiness in agricultural applications by integrating multi-modal data encompassing the combination of image-based phenotypic traits with genomic data. Furthermore, we will expand the scope of this method to encompass a broader range of crops while is imperative for addressing global agricultural diversity.

Subsequent iteration will incorporate a broader spectrum of crop varieties or accessions, extended growth stages, and integrate with more comprehensive phenotyping datasets acquired across diverse field environments, thereby augmenting the platform's applicability across a wider range of agricultural contexts. These enhancements aim to further improve the utility and generalizability of Img2Variety in real-world breeding and phenotyping scenarios.

Furthermore, the Img2Variety framework demonstrate considerable potential for adaptation to additional crop species. Its successful deployment is upon the acquisition of high-resolution RGB imagery of sufficient quality. Optimal results are achieved when imagery captures multiple plant perspectives or distinct growth stages to adequately represent the phenotypic diversity inherent at the accession level. Empirical findings from our experiments indicate that the resolutions of approximately 360 × 480 pixels were sufficient for robust model performance. The complexity of the classification task is influenced by the total number of accessions, while the quality of images per accession directly impacts the model's capacity to learn discriminative varietal characteristics. For example, the model achieved approximately 8 % higher accuracy in rice classification, which utilized 96 images per accession, compared to maize with only 19 images per accession. In scenarios characterized by limited sample availability per accession, GMMA can increase phenotypic diversity through multi-stage and multi-view augmentation. These factors provide practical guidance for applying Img2Variety to other crops, ensuring reliable variety identification.

## Data and code availability

The raw rice dataset is available at https://ngdc.cncb.ac.cn/opia/dataset/datasets?dataId&equals;17, and the corresponding image-based phenotypic traits can be accessed at https://ngdc.cncb.ac.cn/opia/traits. The preprocessed training and testing subsets for the rice dataset can be directly downloaded from the tool page at https://ngdc.cncb.ac.cn/opia/static/tools_data/wgsr_rice.zip. Similarly, the maize dataset is available at https://ngdc.cncb.ac.cn/opia/dataset/datasets?dataId&equals;57, and its corresponding preprocessed training and testing data can be obtained from https://ngdc.cncb.ac.cn/opia/static/tools_data/wgsm_maize.zip. The Python implementation of Img2Variety, which incorporates six baseline convolutional neural network (CNN) models, is publicly available at https://github.com/yongrongc3/Img2Variety.

## Author contribution

Y.R.C. and R.Y.Y. conceptualized the study. Y.R.C., L.L., H.L., and D.M.T. developed the methodology. Y.R.C., D.M.T., and Z.S.W. collected the dataset and performed data preprocessing. Y.R.C. and H.L. wrote the original draft of the manuscript, while Y.R.C., H.L., D.M.T., and S.H.S. reviewed and edited it. S.H.S., D.M.T., and L.L. acquired the funding for the study. S.H.S. and D.M.T. supervised the project.

## Funding

This research was supported by the Strategic Priority Research Program of the 10.13039/501100002367Chinese Academy of Sciences (XDA0460405 to D.T., XDA0450102 to S.S.), 10.13039/501100012166National Key Research and Development Program (2025YFF1207901 to S.S.), Biological Breeding-10.13039/501100018537National Science and Technology Major Project (2022ZD04017 to S.S.), 10.13039/501100004739Youth Innovation Promotion Association of the Chinese Academy of Sciences [Y2021038 to S.S.].

## Declaration of competing interest

The authors declare that they have no known competing financial interests or personal relationships that could have appeared to influence the work reported in this paper.
