# Supplementary material for: Img2Variety: Image-based intraspecific varieties identification across the whole growth period
Source: Plant Phenomics. 2025 Dec 18;8(1):100151. doi: 10.1016/j.plaphe.2025.100151 (PMC13109561; doi:10.1016/j.plaphe.2025.100151)
Supplement: Multimedia component 1 [file mmc1.pdf]

Supplemental Figures

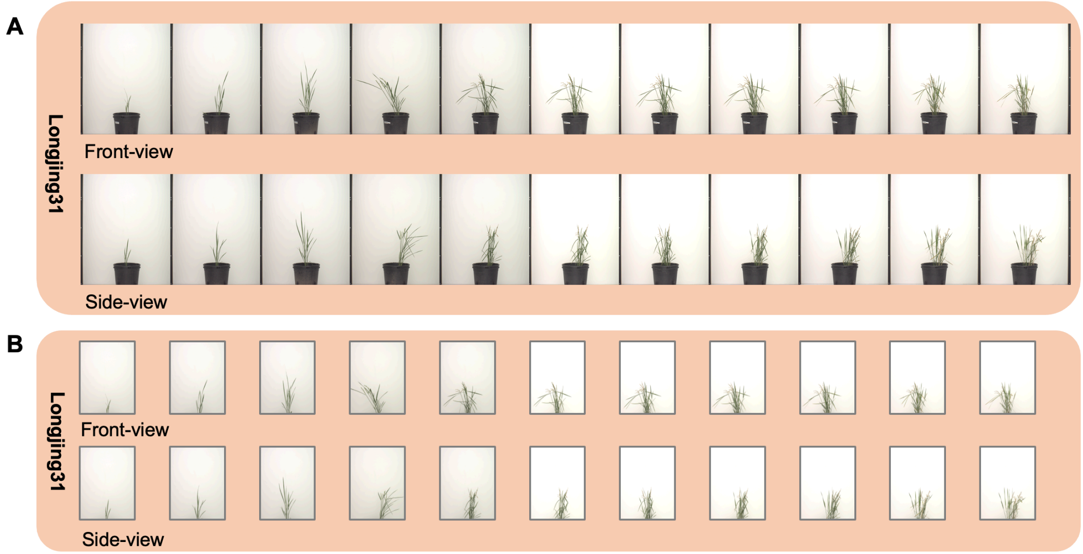

Fig. S1. Images from the rice dataset: (A) Samples of rice accession Longjing31 captured from two different views across all growth stages. (B) Cropped and resized samples of Longjing31.

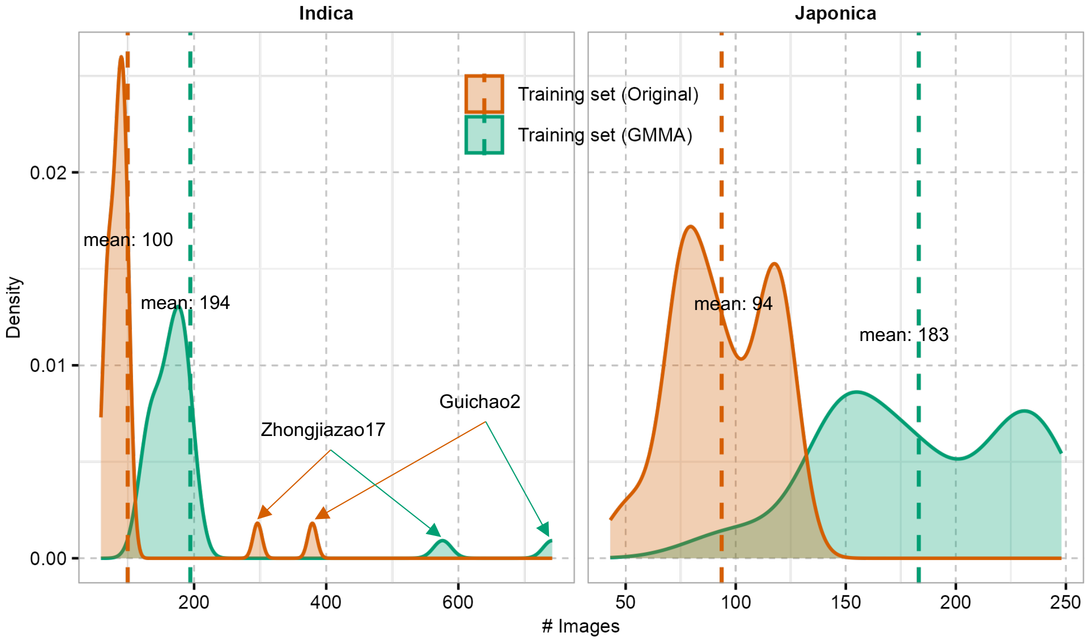

Fig. S2. Density plot of the number of images per accession in the rice training dataset, with two subspecies (indica and japonica). The dashed line represents the average number of images across all rice accessions for original training set and GMMA training set, respectively. In the Indica subgroup, the accessions Zhongjiazao17 and Guichao2 are represented by substantially more samples than other accessions.

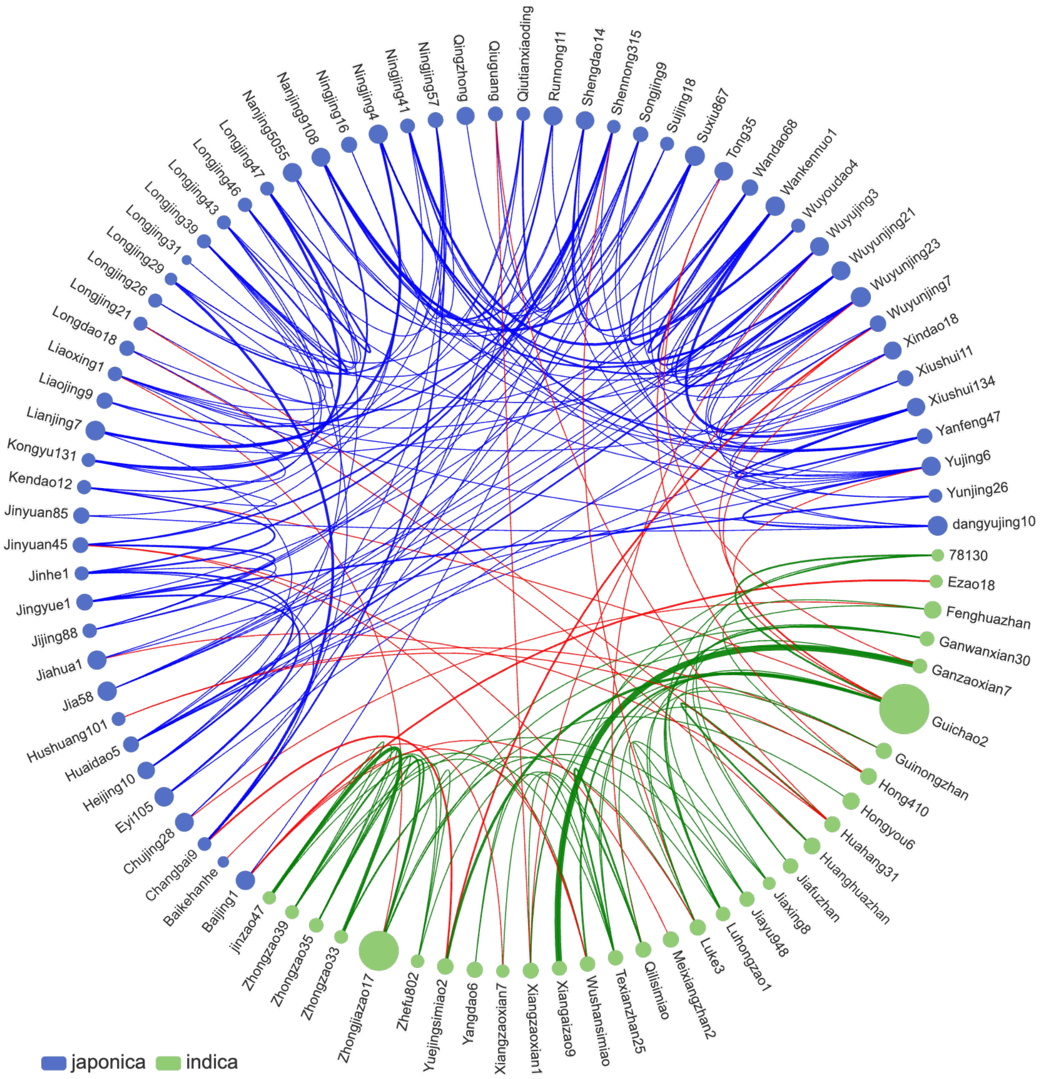

Fig. S3. The network graph depicts prediction errors made by the Img2Variety classification model (backbone DenseNet121) across rice accessions. It reveals misclassification patterns and potential confusion among genetically or visually similar cultivars. Each node represents a rice accession name, with node size proportional to the number of associated image samples. Edges indicate misclassifications between accessions; edge width reflects the frequency of errors. Edge colors represent subspecies relationships: blue for errors between japonica accessions, green for indica accessions, and red for errors between different subspecies (japonica vs. indica).

Supplemental Tables

Table S1 Statistics of rice and maize dataset for varieties identification tasks.

Dataset	Task	Method	# Varieties / Subspecies	Training set	Testing set
Rice	Accessions Identification	-	93	8,895	2,275
		Data augmentation	93	17,391	
	Subspecies Classification	-	Indica	3,185	829
			Japonica	5,710	1,446
		Data augmentation	Indica	6,217	829
			Japonica	11,174	1,446
Maize	Maize Lines Identification	-	224	4307	1292
		Data augmentation	224	8887	

The - indicates dataset without augmenting, data augmentation represents growth-aware multi-view mixed augmentation (GMMA) is added.

Table S2 Performance and resource usage of Img2Variety for identifying 93 rice accessions using six backbone networks.

Backbone network	Method	Accuracy (%)	Precision (%)	Recall (%)	F1-score (%)	Parameters (M)	Peak GPU Memory Usage
VGG19	Baseline	59.4286	59.0421	57.4129	57.1512	139.95	31.41GiB
	Img2Variety	77.8462	77.6164	76.9132	76.8744		
ResNet18	Baseline	68.6593	67.8667	66.8216	66.6284	11.22	8.21GiB
	Img2Variety	83.6484	83.8700	83.0774	83.0653		
MobieNetV2	Baseline	67.1648	66.1941	65.2938	65.1637	2.34	19.29GiB
	Img2Variety	84.7033	84.9183	83.9408	84.0857		
InceptionV3	Baseline	74.4615	73.7549	72.7794	72.7137	25.3	16.42GiB
	Img2Variety	86.0220	85.8388	85.3165	85.2858		
EfficientNet	Baseline	71.9560	71.8903	70.1413	70.2331	4.13	21.55GiB
	Img2Variety	86.2857	86.3840	85.7693	85.7808		
DenseNet121	Baseline	74.9451	74.4531	73.6119	73.4000	7.05	30.81GiB
	Img2Variety	**88.6593**	**88.9655**	**88.3812**	**88.3448**		

Table S3 Performance and resource usage of Img2Variety for 224 maize inbred lines identification using six backbone networks.

Backbone network	Method	Accuracy (%)	Precision (%)	Recall (%)	F1-score (%)	Parameters (M)	Peak GPU Memory Usage
VGG19	Baseline	46.0526	49.7122	44.9740	44.0142	140.49	26.81GiB
	Img2Variety	68.6533	70.7361	67.3170	66.9366		
ResNet18	Baseline	34.4427	40.0106	34.2420	34.1600	11.29	6.81GiB
	Img2Variety	74.6130	76.9354	73.5159	73.2498		
MobieNetV2	Baseline	35.3715	40.2947	34.6643	34.7336	2.51	15.98GiB
	Img2Variety	76.7802	78.4144	75.5397	75.0620		
InceptionV3	Baseline	44.5820	51.0679	43.8354	43.9774	25.57	13.42GiB
	Img2Variety	77.0898	78.8012	75.7063	75.4964		
EfficientNet	Baseline	45.2012	48.8005	44.4342	44.0558	4.29	17.76GiB
	Img2Variety	79.3344	81.6304	77.8286	77.6840		
DenseNet121	Baseline	42.8795	46.2544	42.3275	41.6421	7.18	25.46GiB
	Img2Variety	**79.9536**	**81.3825**	**78.6933**	**78.6164**		

Table S4 Optimal hyperparameter settings of the Img2Variety model using six backbone architectures on the rice accession dataset.

Hyperparameter	VGG19	ResNet18	MobieNet V2	Inception V3	EfficientNet	DenseNet 121
random_seed	42	42	42	42	42	42
batch_size	64	64	64	64	64	64
optimizer (Learning_rate, Momentum)	SGD (0.1, 0.9)	SGD (0.1, 0.9)	SGD (0.1, 0.9)	SGD (0.1, 0.9)	SGD (0.1, 0.9)	SGD (0.1, 0.9)
loss_alpha	0.1	1	0.5	0.5	0.5	0.5
final_fc_dim	(4096, 93)	(512, 93)	(1280, 93)	(2048, 93)	(1280, 93)	(1024, 93)

Table S5 Optimal hyperparameter settings of the Img2Variety model using six backbone architectures on the maize accession dataset.

Hyperparameter	VGG19	ResNet18	MobieNet V2	Inception V3	EfficientNet	DenseNet 121
random_seed	42	42	42	42	42	42
batch_size	64	64	64	64	64	64
optimizer (Learning_rate, Momentum)	SGD (0.1, 0.9)	SGD (0.1, 0.9)	SGD (0.1, 0.9)	SGD (0.1, 0.9)	SGD (0.1, 0.9)	SGD (0.1, 0.9)
loss_alpha	0.1	1	1	0.5	0.9	1
final_fc_dim	(4096, 224)	(512, 224)	(1280, 224)	(2048, 224)	(1280, 224)	(1024, 224)

Table S6 Results of Img2Variety for classifying two rice subspecies.

Backbone network	Method	Accuracy (%)	Precision (%)	Recall (%)	F1-Score (%)
VGG19	Baseline	85.6703	84.6687	84.2238	84.4386
	Img2Variety	94.5055	94.0595	94.8210	94.0708
MobieNetV2	Baseline	85.8901	84.6856	85.0144	84.8428
	Img2Variety	95.9560	95.4221	95.9181	95.6591
ResNet18	Baseline	86.0220	84.8923	84.9637	84.9277
	Img2Variety	96.4835	96.1198	96.3073	96.2121
DenseNet121	Baseline	89.8462	89.2621	88.6926	88.9616
	Img2Variety	96.7912	96.4403	96.6523	96.5444
InceptionV3	Baseline	88.8352	88.1293	87.6399	87.8724
	Img2Variety	96.9231	96.6349	96.7303	96.6822
EfficientNet	Baseline	88.7473	89.1055	86.3869	87.4517
	Img2Variety	**96.9670**	**96.4388**	**97.0994**	**96.7499**

Table S7 Validation and comparison of Img2Variety with traditional machine learning models (identification of 93 rice accessions & classification of rice subspecies).

Tasks	Models	Features	Accuracy (%)	Precision (%)	Recall (%)	F1-Score (%)
Subspecies classification	Naïve Bayes	Hu, HSV	52.66	55.41	55.63	52.58
		Haralick, Hu	60.62	54.83	53.78	53.21
		Haralick, Hu, HSV	60.48	55.38	54.58	54.38
		Haralick, Hu, HSV, LBP	63.12	58.55	57.04	56.94
	Decision Tree	Hu, HSV	71.60	69.33	69.27	69.30
		Haralick, Hu	71.87	69.53	68.99	69.22
		Haralick, Hu, HSV	72.31	70.17	70.39	70.27
		Haralick, Hu, HSV, LBP	74.29	72.26	72.36	72.31
	SVM	Hu, HSV	69.27	69.93	60.18	59.12
		Haralick, Hu	70.55	72.96	61.44	60.61
		Haralick, Hu, HSV	74.11	74.44	67.56	68.42
		Haralick, Hu, HSV, LBP	77.98	78.38	72.79	74.06
	KNN	Hu, HSV	77.23	75.54	74.55	74.96
		Haralick, Hu	75.43	73.60	72.20	72.73
		Haralick, Hu, HSV	79.08	77.66	76.41	76.92
		Haralick, Hu, HSV, LBP	80.35	79.00	78.03	78.45
	**Img2Variety**		**96.97**	**96.44**	**97.10**	**96.75**
Accessions identification	Naïve Bayes	Hu, HSV	5.10	4.49	4.92	3.29
		Haralick, Hu	6.02	6.83	5.62	4.67
		Haralick, Hu, HSV	7.12	7.77	6.67	5.69
		Haralick, Hu, HSV, LBP	7.03	12.54	7.14	6.57
	Decision Tree	Hu, HSV	27.30	26.52	26.59	26.22
		Haralick, Hu	22.46	21.47	21.69	21.21
		Haralick, Hu, HSV	29.10	28.56	28.24	27.99
		Haralick, Hu, HSV, LBP	28.18	27.37	26.84	26.67
	SVM	Hu, HSV	16.48	20.88	12.28	12.74
		Haralick, Hu	20.22	26.15	15.90	17.25
		Haralick, Hu, HSV	26.29	35.31	21.31	23.75
		Haralick, Hu, HSV, LBP	31.21	48.02	26.43	30.13
	KNN	Hu, HSV	28.92	29.80	27.26	26.93
		Haralick, Hu	25.58	25.92	23.60	23.28
		Haralick, Hu, HSV	32.40	32.14	29.79	29.50
		Haralick, Hu, HSV, LBP	32.53	35.38	30.13	30.55
	**Img2Variety**		**88.66**	**88.97**	**88.38**	**88.34**

Table S8 Ablation study on the contributions of Transfer Learning, ACE Loss, and GMMA for rice accession identification.

Backbone network	Transfer Learning	ACE Loss	GMMA	Accuracy (%)	Precision (%)	Recall (%)	F1-Score (%)
VGG19	×	×	×	59.4286	59.0421	57.4129	57.1512
	√	×	×	73.8901	73.5291	72.6412	72.4767
	√	√	×	75.9121	75.6734	74.5981	74.5761
	√	×	√	77.5385	77.5923	76.3336	76.4314
	√	√	√	77.8462	77.6164	76.9132	76.8744
ResNet18	×	×	×	68.6593	67.8667	66.8216	66.6284
	√	×	×	76.4396	76.7184	75.4265	75.5313
	√	√	×	78.1978	77.9783	77.2051	77.2524
	√	×	√	83.3846	83.5703	82.9979	82.9853
	√	√	√	**83.6484**	**83.8700**	**83.0774**	**83.0653**
MobieNet V2	×	×	×	67.1648	66.1941	65.2938	65.1637
	√	×	×	79.2967	79.4427	77.8705	78.1182
	√	√	×	80.0440	80.4251	79.2484	79.4018
	√	×	√	84.7033	84.5712	83.9858	84.0374
	√	√	√	**84.7033**	**84.9183**	**83.9408**	**84.0857**
Inception V3	×	×	×	74.4615	73.7549	72.7794	72.7137
	√	×	×	81.4945	81.3058	80.1945	80.4063
	√	√	×	82.1538	82.2278	80.9663	81.2129
	√	×	√	85.8022	85.8976	85.1267	85.1220
	√	√	√	**86.0220**	**85.8388**	**85.3165**	**85.2858**
EfficientNet	×	×	×	71.9560	71.8903	70.1413	70.2331
	√	×	×	81.4945	82.2701	80.6700	80.8384
	√	√	×	82.3297	83.0014	81.5411	81.4892
	√	×	√	85.4945	85.3333	85.1515	84.9540
	√	√	√	**86.2857**	**86.3840**	**85.7693**	**85.7808**
DenseNet 121	×	×	×	74.9451	74.4531	73.6119	73.4000
	√	×	×	83.9560	84.1444	83.2722	83.2862
	√	√	×	84.2637	83.8383	83.3094	83.2656
	√	×	√	87.8681	88.0179	87.1245	87.2740
	√	√	√	**88.6593**	**88.9655**	**88.3812**	**88.3448**

Table S9 Ablation study on the contributions of Transfer Learning, ACE Loss, and GMMA augmentation for maize lines identification.

Backbone network	Transfer Learning	ACE Loss	GMMA	Accuracy (%)	Precision (%)	Recall (%)	F1-Score (%)
VGG19	×	×	×	46.0526	49.7122	44.9740	44.0142
	√	×	×	61.7647	65.7549	60.4259	60.1370
	√	√	×	65.8669	68.9494	64.4248	64.3869
	√	×	√	68.2663	72.3001	67.2053	67.0997
	√	√	√	68.6533	70.7361	67.3170	66.9366
ResNet18	×	×	×	34.4427	40.0106	34.2420	34.1600
	√	×	×	62.8483	65.2818	61.5420	61.2472
	√	√	×	65.4799	67.3489	64.4334	63.9516
	√	×	√	73.0650	75.3666	71.9112	71.7024
	√	√	√	74.6130	76.9354	73.5159	73.2498
MobieNetV2	×	×	×	35.3715	40.2947	34.6643	34.7336
	√	×	×	63.6223	66.0219	62.7005	62.4444
	√	√	×	67.1053	69.5994	66.1082	65.9392
	√	×	√	73.2972	75.3367	72.0782	71.7275
	√	√	√	76.7802	78.4144	75.5397	75.0620
InceptionV3	×	×	×	44.5820	51.0679	43.8354	43.9774
	√	×	×	68.9628	71.3580	67.5681	67.1603
	√	√	×	71.2848	74.0815	69.7527	69.5540
	√	×	√	76.3932	78.7597	75.0624	75.2459
	√	√	√	77.0898	78.8012	75.7063	75.4964
EfficientNet	×	×	×	45.2012	48.8005	44.4342	44.0558
	√	×	×	71.5944	74.5804	70.3921	70.3640
	√	√	×	73.3746	75.3668	72.2505	71.8975
	√	×	√	78.3282	80.8923	77.1452	76.9284
	√	√	√	79.3344	81.6304	77.8286	77.6840
DenseNet121	×	×	×	42.8795	46.2544	42.3275	41.6421
	√	×	×	68.9628	71.5697	67.8515	67.6514
	√	√	×	72.9876	75.1187	71.8756	71.5142
	√	×	√	77.6316	79.3574	76.4194	76.1010
	√	√	√	**79.9536**	**81.3825**	**78.6933**	**78.6164**

Table S10 Ablation study showing the individual effects of GMMA and ACE on rice accession identification (DenseNet121 baseline).

Backbone network	GMMA	ACE Loss	Transfer Learning	Accuracy (%)	Precision (%)	Recall (%)	F1-Score (%)
DenseNet 121	×	×	×	74.9451	74.4531	73.6119	73.4000
	×	√	×	75.4286	74.9525	74.1810	74.0928
	√	×	×	84.3516	84.9653	83.4659	83.3725
	×	×	√	83.9560	84.1444	83.2722	83.2862
	√	√	√	**88.6593**	**88.9655**	**88.3812**	**88.3448**

Table S11 Ablation study showing the individual effects of GMMA and ACE on maize lines identification (DenseNet121 baseline).

Backbone network	GMMA	ACE Loss	Transfer Learning	Accuracy (%)	Precision (%)	Recall (%)	F1-Score (%)
DenseNet 121	×	×	×	42.8795	46.2544	42.3275	41.6421
	×	√	×	52.6316	57.8963	51.5397	51.5992
	√	×	×	62.2291	65.1003	61.0379	60.6201
	×	×	√	68.9628	71.5697	67.8515	67.6514
	√	√	√	**79.9536**	**81.3825**	**78.6933**	**78.6164**

Table S12 Performance comparison of viewpoint, temporal, and view-stage mixup strategies for rice accession identification under the transfer learning and ACE Loss baseline.

GMMA	Viewpoint	Temporal	View-stage	Accuracy (%)	Precision (%)	Recall (%)	F1-score (%)
# Images	12,416	10,311	12,454				
DenseNet 121	√	×	×	87.4725	87.9140	87.1078	87.1165
	×	√	×	85.8022	86.1308	86.2245	85.3774
	×	×	√	87.8242	87.8718	87.2327	87.2653
	√	√	√	**88.6593**	**88.9655**	**88.3812**	**88.3448**

Table S13 Performance comparison of viewpoint, temporal, and view-stage mixup strategies for maize inbred lines identification under the transfer learning and ACE Loss baseline.

Backbone network	Viewpoint	Temporal	View-stage	Accuracy (%)	Precision (%)	Recall (%)	F1-score (%)
# Images	5290	6781	5430				
DenseNet 121	√	×	×	75.7740	78.3212	74.5072	73.6617
	×	√	×	77.5542	79.6110	76.1590	76.0958
	×	×	√	77.8638	78.5275	76.5831	76.0908
	√	√	√	**79.9536**	**81.3825**	**78.6933**	**78.6164**
